# An Update of Medical Nutrition Therapy in Gestational Diabetes Mellitus

**DOI:** 10.1155/2021/5266919

**Published:** 2021-11-18

**Authors:** Flavia Cristina Vasile, Agnesa Preda, Adela Gabriela Ștefan, Mihaela Ionela Vladu, Mircea-Cătălin Forțofoiu, Diana Clenciu, Ioan Ovidiu Gheorghe, Maria Forțofoiu, Maria Moța

**Affiliations:** ^1^Diab Clinique Craiova, Dolj, Romania; ^2^University of Medicine and Pharmacy of Craiova, Craiova, Dolj, Romania; ^3^Clinical County Emergency Hospital, Craiova, Dolj, Romania; ^4^Department of Diabetes Nutrition and Metabolic Diseases, Calafat Municipal Hospital, Calafat, Dolj, Romania; ^5^Clinical Municipal Hospital “Philanthropy”, Craiova, Romania; ^6^Public Health Department Gorj, Romania

## Abstract

Gestational diabetes mellitus (GDM) is a serious and frequent pregnancy complication that can lead to short and long-term risks for both mother and fetus. Different health organizations proposed different algorithms for the screening, diagnosis, and management of GDM. Medical Nutrition Therapy (MNT), together with physical exercise and frequent self-monitoring, represents the milestone for GDM treatment in order to reduce maternal and fetal complications. The pregnant woman should benefit from her family support and make changes in their lifestyles, changes that, in the end, will be beneficial for the whole family. The aim of this manuscript is to review the literature about the Medical Nutrition Therapy in GDM and its crucial role in GDM management.

## 1. Introduction

The American Diabetes Association (ADA) defines GDM as previously unknown diabetes, diagnosed during the second or third pregnancy trimester [1]. Although GDM is one of the most frequent perinatal complications, its definition, diagnosis criteria, or screening methods did not benefit from a uniform approach from different international organizations. Thus, although the effects of perinatal hyperglycemia were first described by Dr. J.P. Hoet in 1954 [2], the diagnosis criteria and medical care standards for GDM were established only in the last two decades. An important study regarding this matter was HAPO (Hyperglycemia and Adverse Pregnancy Outcomes Study) [3], a study performed on 23 316 pregnant women, whose results led to the establishment of the new criteria for diagnosis of GDM by IADPSG (International Association of Diabetes and Pregnancy Study Groups) in 2010 (Table 1) [4]. Still, an international consensus in this matter does not currently exist [5].

Regarding the screening for GDM, there is no doubt that all pregnant women should be tested for GDM between the 24th and 28th pregnancy weeks, but there is controversy regarding the testing in early pregnancy, during the first prenatal visit. ADA and NICE (National Institute for Health and Care Excellence) recommend screening for early GDM in women with risk factors during their first prenatal visit, using the classical criteria for diagnosing diabetes mellitus (DM) and thus identifying the pregnant women with early GDM and overt diabetes [6]. Other associations, such as FIGO (International Federation of Gynecology and Obstetrics), recommend universal screening for diabetes in early pregnancy, regardless of the presence or absence of the risk factors [7].

Between the 24th and 28^th^ pregnancy weeks, it is recommended to test all pregnant women for GDM.

The main risk factors for GDM are as follows: age > 40 years old, obesity, personal history of GDM or delivery of a macrosomic baby, 1^st^ degree relatives with DM, personal history of polycystic ovary syndrome, some medications (corticosteroids, antipsychotic drugs), multiple births, and race (Asian, Middle East, African-American, and Pacific Islanders) [8].

The last data published by IDF (International Diabetes Federation) in 2019 reported a number of 20 million women (16% of the live births) who presented a form of glucose intolerance during pregnancy, 84% of which being caused by GDM (1 of 6 pregnancies being affected by GDM) [9]. Most cases of hyperglycemia during pregnancy were present in women from low or average developed countries, where the access to medical care is quite limited. These data, however, should be carefully observed, taking into consideration the epidemics of type 2 DM in women of reproductive age and the fact that there is a high number of women with undiagnosed type 2 DM. Taking into consideration the multiple maternal and fetal complications of GDM (Table 2) [9], an early diagnosis and a rapid implementation of medical care standards are crucial. Lifestyle changes must be implemented both during pregnancy and postpartum, through a very close patient-diabetologist-obstetrician relationship. Doctors should make all the efforts to prevent the onset of GDM by controlling the changeable factors (for example obesity), but also, after diagnosis, they should promptly intervene in order to reduce the negative, sometimes catastrophic, effects that this disease may have on the mother and on the offspring.

A special remark should be made on the high risk of pregnant women with GDM to develop type 2 DM in the future [13]. This high risk imposes the indispensability of an appropriate and correct postpartum monitoring, as well as the reduction of modifiable risk factors for an early detection and treatment of possible changes in the glucose metabolism.

The actual pandemic context of SARS-CoV2 infection imposed new guidelines regarding the screening and the postpartum management of GDM. Canadian guidelines proposed HbA1c > 5.7% (39 mmol/mol) or random plasma glucose (RPG) > 200 mg/dl (11.1 mmol/l) as diagnostic criteria for GDM during the COVID-19 pandemic [14]. Australian guidelines diagnose GDM at a FPG > 92 mg/dl (5.1 mmol/mol) and recommend OGTT for levels between 85 and 90 mg/dl (4.7-5 mmol/mol) [15]. In May 2020, RCOG (Royal College of Obstetricians and Gynaecologists) established that women considered being at high risk for GDM should be tested at 28 weeks using HbA1c (GDM: HbA1c > 5.7%) or FPG (GDM: FPG > 95 mg/dl) or RPG (GDM: RPG > 162 mg/dl) [16]. All these measurements reduced the risk of contamination in pregnant women but also failed to detect 57% of cases [17].

Another downside to the pandemic is that pregnant women experience a low well-being state, and this fact has a negative impact on their physical and psychological health. Also, during the COVID-19 outbreak, visits to obstetric triage, gynecologic triage, and ultrasound units decreased by 36.4%, 34.7%, and 18.1%, respectively, according to a cross-sectional study that compared changes in outpatient clinic visits between March-April 2020 and March-April 2019 [18].

The postpartum screening of type 2 diabetes in women with GDM was postponed to 3-6 months after delivery using HbA1c (UK guidelines) [13] or 6-12 months using OGTT (Australian guidelines) [16].

The aim of this paper is to review recent studies and various methods of screening and management of GDM, focusing on the current recommendations concerning the Medical Nutrition Therapy.

## 2. Medical Nutrition Therapy in GDM

Medical Nutrition Therapy (MNT), together with physical exercise and frequent self-monitoring, represents the milestone for the GDM treatment in order to reduce the maternal and fetal complications, on both short and long times. All these interventions involve a strong collaboration between the pregnant woman and the medical care team, based on mutual trust and correct information; therefore, a sustained psychosocial support represents an important part of the therapy. It is a well-known fact that a good emotional state increases the compliance of the pregnant woman to the medical recommendations; stress, anxiety, depression, and nutritional disorders represent some limits that are difficult to overcome during an efficient therapy [19]. MNT, although follows some clear, generally accepted directions, needs to be individualized according to the cultural characteristics, the learning and decisional capacity, and the familial support of every pregnant woman.

Physical activity represents a very important aid for the MNT in GDM, both aerobic exercises (walking, swimming, biking, and prenatal exercises) and mild or moderate resistance exercises, both types being beneficial through increasing the insulin sensitivity. A duration of 30 minutes of physical activity/day is recommended [20]; this duration can be fractioned in 10-minute rounds. Exercises involving lying flat on the back, contact sports, tennis, horse riding, and nautical skiing are not recommended due to the risk of falling or injury. Also, the ones that involve intra-abdominal pressure increase (jumping) are forbidden. In addition, pregnant women should be advised to hydrate accordingly during exercise and to avoid performing physical effort under conditions of high temperature or humidity, when they are hungry or do not feel well [21].

MNT, together with physical exercise, weight control, and implementing a self-control strategy, should begin as soon as possible after diagnosis, namely, in the first week. Pregnant women should be taught to self-monitor fasting and postprandial glucose and to keep a diary where to note down the values of self-measured blood glucose, data on the food, and physical exercise, a diary that should be presented to the medical team. They should also do it in order to identify the individual variations of glycemic values and the factors determining them, thus having the necessary data for taking appropriate decisions regarding their lifestyle changes.

Self-monitoring of blood glucose (SMBG) is an important part of standard diabetes care. Frequent SMBG helps patients understand better the influence that food and exercise have on their blood glucose values, thus increasing their adherence to the treatment plan. SMBG should be performed using capillary blood, and the number of tests required to adequately monitor blood glucose levels depends on several factors: diet, physical activity, type of treatment (diet/insulin), and the risk of hypoglycemia [22].

The German Diabetes Association (DDG) recommends SMBG 4 times/day (fasting, 1 h and 2 h postprandial) in the first two weeks after the diagnosis. If over 50% of the measurements are elevated during these two weeks, we should consider insulin therapy, in which case the patient will need at least 3 tests/day and nocturnal evaluations of the glucose levels. If the patient does not need insulin therapy, SMBG should be performed once/day on a rotation schedule along with two 4-point profiles/week [23].

The glycemic targets recommended for pregnant women with GDM are as follows: fasting plasma glucose < 95 mg/dl (5.3 mmol/l), 1-hour postprandial glucose < 140 mg/dl (7.8 mmol/l), and 2-hour postprandial glucose < 120 mg/dl (6.7 mmol/l). It was shown that reaching and maintaining fasting glucose < 95 mg/dl in the first 2 weeks from implementing MNT are correlated with a reduced possibility of introducing pharmacological treatment [24].

MNT in GDM has the following main objectives: providing the appropriate caloric intake for both mother and fetus, avoiding ketosis, promoting optimal fetal growth, and avoiding the mother's excessive weight gain. The nutrition plan is individualized, taking into consideration the mother's particularities (health state, weight, ethnic, cultural particularities, compliance, etc.), the medical team being the one informing the mother about the risks that this condition may have upon her and the fetus, in order to obtain maximum compliance and adherence. Studies showed that 70-85% of pregnant women diagnosed with GDM obtained and maintained glycemic targets only with MNT [25], its part being of utmost importance for the management of this condition. Although unanimously recognized as the milestone in the treatment of GDM, MNT remains a controversial subject. Even from the first official admission of GDM, various types of diets were proposed, starting from severe restrictions of the carbohydrate intake to more loose diets from this point of view. The data are limited, as the studies were not being conducted on a very large number of women, most studies being also deficient from various points of view (collecting and interpreting the results, data on compliance, etc.).

### 2.1. Caloric Intake

There are limited data that clearly establish the necessary caloric intake and the optimal weight gain in pregnant women with GDM. The Institute of Medicine (IOM) does not recommend weight loss during pregnancy [26], and if the caloric restriction is required, this should be performed in a controlled manner, taking into consideration the fact that severe food restriction may lead to a rapid turn of the body into using fatty acids (FA) and glucose saving [23]. Also, it is well known the negative effect of maternal ketonemia, this being associated with neurological disorders and future cognitive deficits in the baby [27].

Still, we should take into consideration the high percentage of overweight and obese women at their reproductive age (25-40%) [28], most pregnant women with GDM being in this category (40% prevalence of GDM in European obese women) [29]. In this case, maternal hyperglycemia induces an excess of nutrients in the fetal blood stream which leads, through multiple mechanisms, to fetal macrosomia and its multiple complications: mechanical complications during delivery, obesity, and diabetes during the teenage period or adulthood. The results of a recent follow-up study of a cohort from the HAPO Study, performed 11 years after the pregnancy complicated with GDM, identified a high incidence of overweight/obesity in children correlated with the mothers' body mass index (BMI) before pregnancy [10]. Thus, it is very important to intervene on women's lifestyle, conducting information campaigns, and aggressively fight obesity before conception, in order to provide a healthy start in life for future generations.

Therefore, most international organizations (ADA, AND (Academy of Nutrition and Dietetics), and CDA (Canadian Diabetes Association)) recommend that normal and overweight pregnant women should be encouraged in having an adequate weight gain, according to the IOM recommendations. Regarding overweight and obese women, moderate caloric restriction is indicated (a reduction by approx. 30% of the caloric intake prior to pregnancy, taking into consideration that the diet should not have under 1600 kcal/day) (Table 3). No guide recommends weight loss during pregnancy, only to slow down weight gain, thus avoiding maternal ketosis and other side effects on the mother and fetus [30].

### 2.2. Carbohydrate Intake

The idea of carbohydrate (CH) dietary restriction in GDM has its origin even before the insulin era, when it was noted that a severe restriction of CH (8-10% of the total caloric intake) prolonged life in women with type 1 diabetes and reduced the incidence of fetal macrosomia and stillbirth. After the war, starting with the official admission of GDM, this trend was preserved, because of the evidence given by numerous studies that correlated maternal hyperglycemia with fetal macrosomia. In 1990, Jovanovic-Peterson and Peterson [31] proposed that the CH restriction should be considered the first line of treatment in GDM. In the following decades, there was an emphasis on identifying the most appropriate type of diet that provided optimal results, both for the mother and for the fetus. In 2018, Yamamoto et al. [32] published the results of a meta-analysis of 18 studies performed on a total of 1151 women with GDM that showed that a nutritional intervention (change in eating habits including, but not limited to CH restriction) led to the decrease of fasting glucose (by 4 mg/dl), postprandial glucose (by 8 mg/dl), and birth weight (by 171 g).

There are numerous controversies regarding the optimal intake of CH, in terms of quantity and type of CH (Table 4). It raises the question whether the best approach is represented by the CH restriction or by a more

“liberal” diet. There are randomized controlled studies [33] showing that a more “liberal” intake of complex CH provided better control of maternal blood glucose in comparison to the more restrictive CH diets. Although there are numerous studies that have tried to determine which is the optimal quantity of CH that should be consumed by the pregnant women with GDM, a consensus has not been reached, so that, just like in the case of DM, a standard diet cannot be imposed, due to the numerous individual particularities (mother's age, anthropometric parameters, compliance, a correct report in the eating diary, and necessity for insulin), which makes these studies heterogeneous.

At present, the ADA recommendations are that pregnant women with GDM should consume a minimum quantity of 175 g CH/day, representing 35-50% of the total caloric intake. Regarding the CH distribution per meals, there is no evidence from studies highlighting a certain distribution that can be correlated with better results in controlling maternal blood glucose and the effects on the fetus, as well. The quantity and distribution of CH should be made according to the particularities of every pregnant woman: BMI, weight gain during pregnancy, fasting and postprandial glucose values, and presence or absence of ketonemia. Most guides recommend the distribution of CH into 3 main meals (breakfast: 10-15%, lunch: 20-30%, and dinner: 30-40%) and 3 small snacks (5-10% of the total CH intake). The CH intake during breakfast should be reduced to 15-30 g, taking into consideration the morning peak of cortisol secretion, which explains why most pregnant women with GDM present high blood glucose values after breakfast. In the last decades, the emphasis went more and more on the use of low glycemic index (GI) CH. The glycemic index is a value assigned to foods that defines their impact on postprandial glucose values [34].

The consumption of low GI food is considered to be associated with a lower risk of fetal macrosomia, due to lower postprandial glycemic values. This hypothesis was also the conclusion of a meta-analysis including 5 randomized controlled studies on a total number of 302 pregnant women [35]. Also, a study performed in China on 140 pregnant women [36], which randomized the subjects into a group that followed a diet based on low GI food and another group that followed a diet based on high GI food, with an equal intake of CH in the two groups, showed an extra reduction of fasting glucose (-3.7% in the first group in comparison to -1.2% in the second group) and of postprandial glucose (-19-22% in the first group versus –7-12% in the second group). All these data suggest that diets based on food with a low GI improve the glycemic profile of mothers with GDM and reduce the risk of fetal macrosomia. This aspect could also be used in deciding a menu for breakfast, especially in women who have difficulties in controlling postprandial glucose during this time of the day.

Regarding the fiber intake, ADA recommends an intake of 28 g/day, coming mainly from cereals, fruits, and vegetables, due to their well-known positive effect on the control of postprandial glucose. Studies that investigated the effect of high fiber intake diets (80 g/day) reported a low compliance of pregnant women to this type of diet (40-60%), due to the gastrointestinal side effects [37]. Recently, a meta-analysis highlighted that the risk for fetal macrosomia was reduced in pregnant women with GDM who had a diet based on low GI foods and high fiber intake, in comparison to those having a diet with low GI foods and low fiber intake [35].

### 2.3. Protein Intake

During pregnancy, an appropriate protein intake is crucial in order to promote fetal growth and development. There is no evidence from studies indicating a particularity of pregnant women with GDM, neither regarding the protein quantity recommended during pregnancy nor their type. ADA recommends a protein intake of a minimum 71 g/day in pregnant women with GDM for all stages of pregnancy. Recently, a study that used the minimally invasive indicator amino acid oxidation method established the protein requirements to increase from 1.2 g/kg/day at 16 weeks of pregnancy to 1.52 g/kg/day at 36 weeks [38]. Thus, the recommendations regarding the protein and amino acid intake should vary according to the gestational age, in order to adequately fulfill the increasing needs of the mother and the fetus.

The main protein sources are represented by low-fat white and red meat, eggs, soya, nuts, and vegetables. Animal products should be very well and healthy cooked. A special remark should be made regarding the fish consumption. Fish and seafood represent an extremely rich source of proteins, iron, and omega-3, vital for the development of the fetus brain. Nonetheless, these species commonly come from mercury-polluted water, this leading to intoxications, with serious effects on the mother and fetus (neurological damage, cognitive, attention, memory, and language problems) [39, 40].

### 2.4. Lipid Intake

In GDM, the restriction of the CH intake may lead to the tendency of pregnant women to consume a higher quantity of lipids. This behaviour was shown in numerous studies to have negative consequences for the health of both mother and fetus. First of all, the high level of free fatty acids (FFA) increases insulin resistance. Moreover, a high level of triglycerides (TG) and FFA in the maternal serum was correlated with fetal macrosomia, due to TG hydrolysis and the FFA transport through the placenta to the fetus where it contributes to an excessive fetal growth. A study [41] performed on 34 pregnant women following the diet DASH (Dietary Approaches to Stop Hypertension) (65% CH, 18% lipids) for 4 weeks highlighted the following beneficial effects: decrease of glycated haemoglobin (HbA1c), of systolic blood pressure, of seric lipids, and of oxidative stress and improvement of insulin resistance.

At present, the IOM recommendations indicate a lipid intake of 20-35% of the total caloric intake. German guides recommend that a percentage of 30-35% from the caloric intake should be covered by lipids, specifying that obese women should prefer low-fat food [21]. The saturated FA and Trans FA should be reduced as much as possible, down to 7% from the caloric intake. As such, pregnant women are advised to choose meat with a fat content below 10%, as well as low-fat dairy products. The remaining percentage is divided between monounsaturated fatty acids (MUFA) (olive oil, nuts, peanut, nuts, and avocado), polyunsaturated fatty acids (PUFA), omega-3 (fish, fish oil, and flax oil), and omega-6 (soya oil, sunflower, rape, and corn oil). The report between these three types of FA is not clearly defined in pregnant women with GDM. There were studies highlighting the fact that supplementing the diet with PUFA n-3 reduced fetal macrosomia [42]; still, additional studies are necessary to clearly establish the quantity in which these FA should be found in the diet of pregnant women with GDM (Table 5).

### 2.5. Vitamin and Mineral Intake

Pregnancy represents a time when women need a high intake of vitamins and minerals, in order to ensure both their needs and the ones of their babies. In a varied and correct diet, all their needs should be covered. In practice, though, most of the time, supplements of vitamins and minerals are used in order to ensure the high necessary intake. Folic acid is essential for the synthesis of nucleic acid, being vital for fetal growth. Supplementing the diet with folic acid before conception and during the first 12 weeks of pregnancy considerably reduced the percentage of pregnancies with neuronal tube defects in children. Supplements with folic acid are recommended in a dose of 5 mg/day, 3 months before conception, reducing the dose down to 0.4-1 mg/day starting from the 12^th^ week of gestation [30].

Vitamins C and E are known as strong antioxidants and are very important in the diet of all pregnant women, being the well-known fetotoxic role of the oxidative stress. Although there were theories according to which vitamin C and E supplements could reduce preeclampsia incidence (knowing the role that the oxidative stress plays in this condition), these facts were not clearly defined by studies. Recently, though, a meta-analysis that collected data from the studies performed on a total of 249 975 pregnant women showed a clear relation between the administration of supplements with vitamin D and multivitamins and the reduction of the risk for preeclampsia, a correlation that was not observed in the case of administrating only vitamins C and E [43].

### 2.6. 25-Hydroxyvitamin D

There are some studies that establish a correlation between vitamin D deficit and the onset of GDM, but none of them can find a clear causality relation. In a randomized study conducted by Asemi et al. [44], 54 pregnant women with GDM received either placebo or 2 doses of 50 000 UI vitamin D for a period of 6 weeks. Pregnant women who received the supplements of vitamin D presented a statistically significant decrease of fasting glucose and insulin resistance assessed through the HOMA IR index. A recent meta-analysis [45] that included 6 randomized studies concluded that the administration of vitamin D supplements led to the improvement of insulin sensitivity, still not to the reduction of fasting glucose or HbA1c. Additional studies are required to clearly establish the connection between vitamin D supplements and the prevention or treatment of GDM. At present, IOM recommends a dose of 5 *μ*g/day while in the North European countries, where the seric concentrations of 25(OH)D are low during winter; 10 *μ*g/day is recommended [46].

During pregnancy, supplements with vitamin A are contraindicated.

The calcium necessary is high during pregnancy. At present, an intake of 900-1000 mg calcium/day is recommended [30].

According to CDC (Centers for Disease Control and Prevention), the iron necessary during pregnancy is of 27 mg/day. This may be ensured through a correct diet, iron supplements being required only in the case of an iron-deficiency anemia. The subject of iron supplementing or of an excess iron intake is a controversial one in GDM. The results of a prospective study performed on 3 158 pregnant women identified a 50% higher risk for GDM in pregnant women who had an excess of heme iron (mainly found in chicken meat and red meat) [47].

### 2.7. Sugar Substitutes

The intake of sugar substitutes by pregnant women with GDM is allowed, within the limits set by the FDA (Food and Drug Administration) [48], the key word in their case being moderation. Safe sugar substitutes are the following ones: aspartame (except for women with phenylketonuria), sucralose, neotame, advantame, xylitol, sorbitol (may have gastrointestinal side effects), and stevia. Regarding saccharine, although the FDA considers it safe for consumption in the general population, there are countries where it was prohibited as it may cross the placenta and stay for a long time in the fetus tissues, the side effects on the latter one being unknown (Table 6).

Regarding the intake of coffee, alcohol, and smoking, pregnant women with GDM are to follow the general recommendations during pregnancy: alcohol is strictly prohibited (risk for fetal alcoholic syndrome), caffeine intake should be reduced to a maximum of 200 mg/day, and smoking should be discouraged (Table 7).

## 3. New Research Directions for DM Prevention

### 3.1. Plant-Based Diets

Scientific evidence suggests that plant-based diets can prevent type 2 diabetes by decreasing gastric emptying, improving insulin sensitivity, and increasing insulin secretion [50]. Lately, there are new evidences that suggest that a diet based on plant-derived food may have a positive impact on GDM also by enhancing antioxidant compounds [51]. Women with GDM have increased levels of oxidative stress and inflammatory markers (tumor necrosis factor alpha (TNF-*α*), interleukin 6 (IL-6), and C-reactive protein (CRP)) that could be modulated by diets based on plant-derived food such as the Mediterranean Diet. This diet is based on: vegetables, fruits, nuts, seeds, oils, beans, and whole grains.

Taking into consideration the role that some cytokines, especially IL-6, play in the respiratory syndrome of COVID-19 and the fact that the Mediterranean Diet can modulate TNF-*α*, IL6, and CRP, there are reasons to believe that this diet can reduce the risk of GDM and improve the immune response in COVID-19 pneumonia [52]. This topic is of particular interest for the researchers, but further studies are required.

### 3.2. Myo-Inositol

Myo-inositol and D-chiro-inositol are the most studied representatives of the inositol family, molecules with an important role played in obtaining and maintaining a healthy pregnancy. These are involved in the cellular energetic metabolism, follicular maturation, and cellular motility. In the last years, myo-inositol was studied in relation to the favorable impact on fertility, as well as for the prevention of certain complications during pregnancy, such as fetal neural tube birth defects or maternal GDM [53]. The role played by the two inositols in the intracellular transmission of the insulin signal was identified for the first time by Larner et al. [54]. Ever since, more and more researchers have been investigating the favorable effects of myo-inositol administration in patients with DM and insulin-resistance, with favorable obtained results. Regarding the positive effects of the administration of myo-inositol supplements for GDM prevention, there are already a series of studies including pregnant women or women during their fertile age, special results being obtained both in women with glucidic metabolism disorders and in those with normal glucose tolerance still with risk factors. In 2012, D'Anna et al. [55] published the results of a study performed on 98 women diagnosed with polycystic ovaries, to whom either 4000 mg myo-inositol/day or 1500 mg metformin/day was administered until the pregnancy onset. The GDM incidence in the group of women treated with myo-inositol was 17.4%, compared to 54% in the group treated with metformin. In 2013, Matarrelli et al. [56] published the results of a study on 73 pregnant women or those who wanted to conceive, with FBG values between 92 mg/dl and 126 mg/dl. The results were overwhelming: the GDM incidence was 6% in the group treated with 4000 mg myo-inositol/day, in comparison to 71% in the control group, the necessity of introducing insulin therapy being 3% in the group treated with myo-inositol compared to 21% in the control group, while neonatal hypoglycemia was 0% in the group treated with myo-inositol, compared to 26% in the control group. The reduction of GDM incidence was also quoted by numerous other studies performed on pregnant women with obesity/overweight/family history of DM or glucidic metabolism change, all these results being in favor for the administration of myo-inositol supplements in women at risk, in order to prevent GDM [57–60].

Pregnancy represents a special time in the life of every family, and it should be seen as an opportunity to implement a healthy lifestyle and to break the vicious circle of unhealthy choices and obesity and metabolic syndrome transmitted from one generation to another. In this period of time, families are more motivated and committed to changes and healthy choices. This is the right moment when the medical team may implement efficient prevention strategies for fighting against the epidemics of obesity and DM.

## 4. Conclusions

GDM is the most common medical complication of pregnancy. Reaching an international consensus regarding the screening, management, and follow-up for women with GDM is of extreme importance in order to prevent the short and long-time complications.

Women with GDM should receive MNT as soon as possible after the diagnosis, but prevention is of utmost importance among pregnant women and women that are trying to conceive.

In this new COVID Era, anxiety and fear of contamination, as well as the limited access to maternal care, led to many cases of undiagnosed GDM, with significant implications for the future.

Further studies are needed in order to evaluate the benefits of different types of diets for women with GDM.

## Figures and Tables

**Table 1:** Criteria of diagnosis for GDM- (OGTT with 75 g glucose)—adapted after [4].

Gestational diabetes	Fasting plasma glucose (FPG)	1 h plasma glucose (OGTT)	2 h plasma glucose (OGTT)	Observations
	≥92 mg/dl (5.1 mmol/l)	≥180 mg/dl (10 mmol/l)	≥153 mg/dl (8.5 mmol/l)	A pathological value may support the diagnosis for GDM

**Table 2:** Effects of maternal hyperglycemia on the mother and offspring—adapted after [8].

Maternal risks^∗^	Short term	(i) Preeclampsia (ii) High blood pressure (iii) Premature birth (iv) Caesarean section (v) Polyhydramnios (vi) Postpartum bleeding (vii) Infection
	Long term	(i) GDM in the next pregnancies (ii) Diabetes Mellitus (5-6.5%, 6 months after birth) [10] (iii) Metabolic syndrome (iv) Cardiovascular/renal disease
Fetal/newborn baby risks	Short term	(i) Prematurity (especially in the case of important maternal hyperglycemia) [11] (ii) Macrosomia (especially in the case of important maternal hyperglycemia) [12] (iii) Fetal injury at birth (iv) Hypoglycemia (v) Polycythemia (vi) Cardiac malformations (hypertrophic cardiopathy) (vii) Stillbirth
	Long term	High risk of DM, obesity/overweight

^∗^There is a clear relation of causality between the levels of hyperglycemia and the complications occurring in the mother and the offspring.

**Table 3:** The caloric intake of pregnant women with GDM according to DDG-DGGG (German Diabetes Association and German Association for Gynaecology and Obstetrics) [23].

BMI prior to pregnancy (kg/m^2^)	Caloric intake (kcals/kg/day)
<18.5 (underweight)	35–40
18.5–24.9 (normal weight)	30–34
25–29.9 (overweight)	25–29
≥30 (obesity)	Maximum 24 kcals/kg/day or a reduction of 30–33% of the prior caloric intake

**Table 4:** Glycemic index of various foods—adapted after [10].

Low GI (<55)	Medium GI (55-69)	High GI (70-100)
Cauliflower, leek, cabbage, beans, strawberries, peaches, apples, plums, pineapple, milk, yogurt, rye bread, whole grain pasta	Bananas, jam, honey, couscous, pizza, polenta, whole flour bread	Chocolate, donuts, potatoes, white flour, corn flakes

**Table 5:** Recommended carbohydrate, protein, and lipid intake in GDM.

Macronutrients	% caloric intake
Carbohydrates	35–50% (minimum 175 g/day) (ADA)
Proteins	71 g/day (ADA)
Lipids	20–35% (IOM) 30–35% (DDG)

**Table 6:** Sugar substitutes, acceptable daily intake: adapted after [48].

Sweetener	Examples of brand names containing sweetener	Acceptable daily intake (mg/kg body weight/day)
Acesulfame potassium (Ace-K)	SweetOne® Sunett®	15
Advantame		32.8
Aspartame	Nutrasweet® Equal® Sugar Twin®	50
Neotame	Newtame®	0.3
Saccharin	Sweet and Low® Sweet Twin® Sweet'N Low® Necta Sweet®	15
Sucralose	Splenda®	5
Certain high-purity steviol glycosides purified from the leaves of *Stevia rebaudiana* (Bertoni) Berton	Truvia® PureVia® Enliten®	4

**Table 7:** Caffeine content of different beverages: adapted after [49].

Drink	Average amount of caffeine (mg)
Brewed coffee 220 ml	135 (80-200)
Instant coffee 220 ml	75
Instant tea 220 ml	26-36
Soft drinks (Cola) 330 ml	35

## References

[1] American Diabetes Association (2021). 2. Classification and Diagnosis of Diabetes:Standards of Medical Care in Diabetes—2021. *Diabetes Care*.

[2] Hoet J. P., Lukens F. D. W. (1954). Carbohydrate metabolism During pregnancy. *Diabetes*.

[3] HAPO Study Cooperative Research Group, Metzger B. E., Lowe L. P. (2008). Hyperglycemia and adverse pregnancy outcomes. *The New England Journal of Medicine*.

[4] International Association of Diabetes and Pregnancy Study Groups Consensus Panel Metzger BE, Gabbe S. G., Persson B. (2010). International association of diabetes and pregnancy study groups recommendations on the diagnosis and classification of hyperglycemia in pregnancy. *Diabetes Care*.

[5] Cheung N. W., Moses R. G. (2018). Gestational diabetes mellitus: is it time to reconsider the diagnostic criteria?. *Diabetes Care*.

[6] Mission J. F., Catov J., Deihl T. E., Feghali M., Scifres C. (2017). Early pregnancy diabetes screening and Diagnosis. *Obstetrics and Gynecology*.

[7] Hod M., Kapur A., Sacks D. A. (2015). The International Federation of Gynecology and Obstetrics (FIGO) initiative on gestational diabetes mellitus: a pragmatic guide for diagnosis, management, and care#. *International Journal of Gynecology & Obstetrics*.

[8] Queensland clinical guidelines (2021). *Gestational diabetes mellitus (GDM). Guideline No. MN21.33-V4-R26*.

[9] International Diabetes Federation (2019). *IDF Diabetes Atlas, 9^th^ Edn, Brussels*.

[10] Lowe W. L., Scholtens D. M., Lowe L. P. (2018). Association of gestational diabetes with maternal disorders of glucose metabolism and childhood adiposity. *Journal of the American Medical Association*.

[11] Battarbee A., Venkatesh K., Aliaga S., Boggess K. A. (2020). The association of pregestational and gestational diabetes with severe neonatal morbidity and mortality. *Journal of Perinatology*.

[12] Berger H., Gagnon R., Sermer M. (2019). Guideline No. 393-Diabetes in Pregnancy. *Journal of Obstetrics and Gynaecology Canada*.

[13] Corrado F., D’Anna R., Laganà A. S., di Benedetto A. (2014). Abnormal glucose tolerance later in life in women affected by glucose intolerance during pregnancy. *Journal of Obstetrics and Gynaecology*.

[14] Yamamoto J. M., Donovan L. E., Feig D. S., Berger H. (2020). Urgent Update—Temporary Alternative Screening Strategy for Gestational Diabetes Screening during the COVID-19 Pandemic. A Joint Consensus Statement from the Diabetes Canada Clinical Practice Guidelines Steering Committee and the Society of Obstetricians and Gynecologists of Canada.

[15] Australasian Diabetes in Pregnancy Society (2020). *Diagnostic testing for gestational diabetes mellitus (GDM) during the COVID-19 pandemic: antenatal and postnatal testing advice*.

[16] Thangaratinam S., Saravanan P., Huda M. S. B., Murphy H. R., Williamson C. (2020). *Guidance for Maternal Medicine in the Evolving Coronavirus (COVID-19) Pandemic. Information for Healthcare Professionals; Version 2.1.*.

[17] van-de-l’Isle Y., Steer P. J., Watt Coote I., Cauldwell M. (2021). Impact of changes to national UK guidance on testing for gestational diabetes screening during a pandemic: a single-centre observational study. *BJOG*.

[18] Justman N., Shahak G., Gutzeit O. (2020). Lockdown with a price: the impact of the COVID-19 pandemic on prenatal care and perinatal outcomes in a tertiary care center. *The Israel Medical Association Journal*.

[19] National Diabetes Services Scheme Gestational diabetes: caring for yourself and your baby. https://www.ndss.com.au.

[20] Duarte-Gardea M. O., Gonzales-Pacheco D. M., Reader D. M. (2018). Academy of nutrition and dietetics gestational diabetes evidence-based nutrition practice guideline. *Journal of the Academy of Nutrition and Dietetics*.

[21] United States Department of Health Human and Services Physical Activity Guidelines for Americans 2nd edition. https://health.gov.

[22] Negrato C. A., Zajdenverg L. (2012). Self-monitoring of blood glucose during pregnancy: indications and limitations. *Diabetology and Metabolic Syndrome*.

[23] Kleinwechter H., Schäfer-Graf U., Bührer C. (2014). German Diabetes Association; German Association for Gynaecology and Obstetrics. Gestational diabetes mellitus (GDM) diagnosis, therapy and follow-up care: Practice Guideline of the German Diabetes Association (DDG) and the German Association for Gynaecologyand Obstetrics (DGGG). *Experimental and Clinical Endocrinology & Diabetes*.

[24] Barnes R. A., Wong T., Ross G. P. (2016). A novel validated model for the prediction of insulin therapy initiation and adverse perinatal outcomes in women with gestational diabetes mellitus. *Diabetologia*.

[25] Mayo K., Melamed N., Vandenberghe H., Berger H. (2015). The impact of adoption of the International Association of Diabetes in Pregnancy Study Group criteria for the screening and diagnosis of gestational diabetes. *American Journal of Obstetrics and Gynecology*.

[26] Moreno-Castilla C., Mauricio D., Hernandez M. (2016). Role of medical nutrition therapy in the management of gestational diabetes mellitus. *Current Diabetes Reports*.

[27] Qian M., Wu N., Li L. (2020). Effect of elevated ketone body on maternal and infant outcome of pregnant women with abnormal glucose metabolism during pregnancy. *Diabetes Metab Syndr Obes.*.

[28] Devlieger R., Benhalima K., Damm P. (2016). Maternal obesity in Europe: where do we stand and how to move forward?: A scientific paper commissioned by the European Board and College of Obstetrics and Gynaecology (EBCOG). *European Journal of Obstetrics, Gynecology, and Reproductive Biology*.

[29] on behalf of the DALI Core Investigator group, Egan A. M., Vellinga A. (2017). Epidemiology of gestational diabetes mellitus according to IADPSG/WHO 2013 criteria among obese pregnant women in Europe. *Diabetologia*.

[30] Blumer I., Hadar E., Hadden D. R. (2013). Diabetes and pregnancy: an endocrine society clinical practice guideline. *The Journal of Clinical Endocrinology and Metabolism*.

[31] Jovanovic-Peterson L., Peterson C. M. (1990). Dietary manipulation as a primary treatment strategy for pregnancies complicated by diabetes. *Journal of the American College of Nutrition*.

[32] Yamamoto J. M., Kellett J. E., Balsells M. (2018). Gestational diabetes mellitus and diet: a systematic review and meta-analysis of randomized controlled trials examining the impact of modified dietary interventions on maternal glucose control and neonatal birth weight. *Diabetes Care*.

[33] Hernandez T. L., van Pelt R. E., Anderson M. A. (2016). Women with gestational diabetes mellitus randomized to a higher-complex carbohydrate/low-fat diet manifest lower adipose tissue insulin resistance, inflammation, glucose, and free fatty acids: a pilot study. *Diabetes Care*.

[34] Atkinson F. S., Foster-Powell K., Brand-Miller J. C. (2008). International tables of glycemic index and glycemic load Values: 2008. *Diabetes Care*.

[35] Wei J., Heng W., Gao J. (2016). Effects of low glycemic index diets on gestational diabetes mellitus: a meta-analysis of randomized controlled clinical trials. *Medicine (Baltimore)*.

[36] Hu Z. G., Tan R. S., Jin D., Li W., Zhou X. Y. (2014). A low glycemic index staple diet reduces postprandial glucose values in Asian women with gestational diabetes mellitus. *Journal of Investigative Medicine*.

[37] Reece E. A., Hagay Z., Gay L. J. (1995). A randomized clinical trial of a fiber-enriched diabetic diet vs. the standard American Diabetes Assocation-recommended diet in the management of diabetes mellitus in pregnancy. *J. Matern. Fetal Investig.*.

[38] Elango R., Ball R. O. (2016). Protein and amino acid requirements during pregnancy. *Advances in nutrition (Bethesda, MD)*.

[39] Swain E. B., Jakus P. M., Rice G. (2007). Socioeconomic consequences of mercury use and pollution. *Ambio*.

[40] Storelli M. M., Marcotrigiano G. O. (2000). Fish for human consumption: risk of contamination by mercury. *Food Additives and Contaminants*.

[41] Asemi Z., Tabassi Z., Samimi M., Fahiminejad T., Esmaillzadeh A. (2013). Favourable effects of the Dietary Approaches to Stop Hypertension diet on glucose tolerance and lipid profiles in gestational diabetes: a randomised clinical trial. *The British Journal of Nutrition*.

[42] Yessoufou A., Nekoua M. P., Gbankoto A., Mashalla Y., Moutairou K. (2015). Beneficial effects of omega-3 polyunsaturated fatty acids in gestational diabetes: consequences in macrosomia and adulthood obesity. *Journal Diabetes Research*.

[43] Fu Z. M., Ma Z.-z., Liu G.-j., Wang L.-l., Guo Y. (2018). Vitamins supplementation affects the onset of preeclampsia. *Journal of the Formosan Medical Association*.

[44] Asemi Z., Hashemi T., Karamali M., Samimi M., Esmaillzadeh A. (2013). Effects of vitamin D supplementation on glucose metabolism, lipid concentrations, inflammation, and oxidative stress in gestational diabetes: a double-blind randomized controlled clinical trial. *The American Journal of Clinical Nutrition*.

[45] Akbari M., Mosazadeh M., Lankarani K. (2017). The effects of vitamin D supplementation on glucose metabolism and lipid profiles in patients with gestational diabetes: a systematic review and meta-analysis of randomized controlled trials. *Hormone and Metabolic Research*.

[46] Yaktine A. L., Rasmussen K. M., Youth F., National Research Council; Institute of Medicine; Board on Children; Committee to Reexamine IOM Pregnancy Weight Guidelines (2009). *Weight Gain During Pregnancy: Reexamining the Guidelines (2009); Rasmussen, K.M., Yaktine, A.L., Eds.*.

[47] Qiu C., Zhang C., Gelaye B., Enquobahrie D. A., Frederick I. O., Williams M. A. (2011). Gestational diabetes mellitus in relation to maternal dietary heme iron and nonheme iron intake. *Diabetes Care*.

[48] US Food and Drug Administration (2018). *Additional information about high-intensity sweeteners permitted for use in food in the United States, February*.

[49] Moderate caffeine consumption during pregnancy (2010). Committee Opinion No. 462: Moderate Caffeine Consumption During Pregnancy. *Obstetrics and Gynecology*.

[50] Pistollato F., Sumalla Cano S., Elio I., Masias Vergara M., Giampieri F., Battino M. (2015). Plant-based and plant-rich diet patterns during gestation: beneficial effects and possible shortcomings. *Advances in Nutrition*.

[51] Schiattarella A., Lombardo M., Morlando M., Rizzo G. (2021). The impact of a plant-based diet on gestational diabetes: a review. *Antioxidants (Basel)*.

[52] Fedullo A. L., Schiattarella A., Morlando M. (2021). Mediterranean diet for the prevention of gestational diabetes in the COVID-19 era: implications of Il-6 in diabesity. *Molecular sciences*.

[53] Gambioli R., Forte G., Buzzaccarini G., Unfer V., Laganà A. S. (2021). Myo-inositol as a key supporter of fertility and physiological gestation. *Pharmaceuticals (Basel)*.

[54] Larner J., Brautigan D. L., Thorner M. O. (2010). D-chiro-inositol glycans in insulin signaling and insulin resistance. *Molecular Medicine*.

[55] D’Anna R., di Benedetto V., Rizzo P. (2012). Myo-inositol may prevent gestational diabetes in PCOS women. *Gynecological Endocrinology*.

[56] Matarrelli B., Vitacolonna E., D’angelo M. (2013). Effect of dietary myo-inositol supplementation in pregnancy on the incidence of maternal gestational diabetes mellitus and fetal outcomes: a randomized controlled trial. *The Journal of Maternal-Fetal & Neonatal Medicine*.

[57] D'Anna R., Scilipoti A., Giordano D. (2013). Myo-inositol supplementation and onset of gestational diabetes mellitus in pregnant women with a family history of type 2 diabetes: a prospective, randomized, placebo-controlled study. *Diabetes Care*.

[58] D'Anna R., di Benedetto A., Scilipoti A. (2015). Myo-inositol supplementation for prevention of gestational diabetes in obese pregnant women: a randomized controlled trial. *Obstetrics and Gynecology*.

[59] Santamaria A., di Benedetto A., Petrella E. (2016). Myo-inositol may prevent gestational diabetes onset in overweight women: a randomized, controlled trial. *The Journal of Maternal-Fetal & Neonatal Medicine*.

[60] Santamaria A., Alibrandi A., Di Benedetto A. (2018). Clinical and metabolic outcomes in pregnant women at risk for gestational diabetes mellitus supplemented with myo-inositol: a secondary analysis from 3 RCTs. *American Journal of Obstetrics and Gynecology*.

